# 9-(2-Ethyl­phenoxy­carbon­yl)-10-methyl­acridinium trifluoro­methane­sulfonate

**DOI:** 10.1107/S1600536810008950

**Published:** 2010-03-13

**Authors:** Damian Trzybiński, Karol Krzymiński, Artur Sikorski, Piotr Malecha, Jerzy Błażejowski

**Affiliations:** a Faculty of Chemistry, University of Gdańsk, J. Sobieskiego 18, 80-952 Gdańsk, Poland

## Abstract

In the crystal structure of the title compound, C_23_H_20_NO_2_^+^·CF_3_SO_3_^−^, the cations form inversion dimers through π–π inter­actions between the acridine ring systems. These dimers are further linked by C—H⋯π inter­actions. The cations and anions are connected by C—H⋯O and C—F⋯π inter­actions. The acridine and benzene ring systems are oriented at a dihedral angle of 20.8 (1)°. The carboxyl group is twisted at an angle of 66.2 (1)° relative to the acridine skeleton. The mean planes of adjacent acridine units are parallel in the lattice.

## Related literature

For general background to 9-(phenoxy­carbon­yl)-10-alkyl­acridinium salts, see: Brown *et al.* (2009 ▶); Rak *et al.* (1999 ▶); Roda *et al.* (2003 ▶); Zomer & Jacquemijns (2001 ▶). For related structures, see: Sikorski *et al.* (2005*a* ▶,*b* ▶). For inter­molecular inter­actions, see: Bianchi *et al.* (2004 ▶); Dorn *et al.* (2005 ▶); Hunter *et al.* (2001 ▶); Steiner (1999 ▶); Takahashi *et al.* (2001 ▶). For the synthesis, see: Niziołek *et al.* (2008 ▶); Sato (1996 ▶).
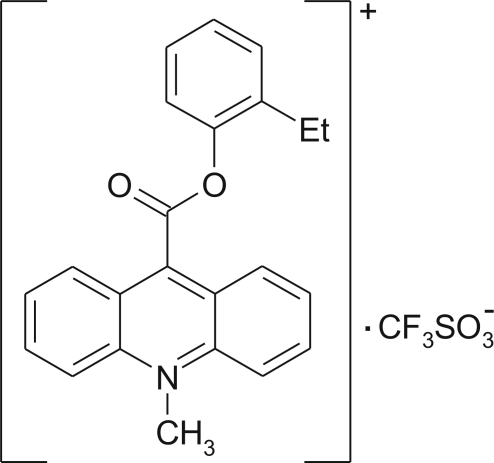


## Experimental

### 

#### Crystal data

C_23_H_20_NO_2_^+^·CF_3_O_3_S^−^
*M* *_r_* = 491.47
Triclinic, 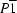

*a* = 9.8519 (4) Å
*b* = 10.9533 (4) Å
*c* = 11.7805 (4) Å
α = 104.379 (3)°
β = 101.475 (3)°
γ = 109.983 (3)°
*V* = 1099.61 (7) Å^3^
*Z* = 2
Mo *K*α radiation
μ = 0.21 mm^−1^
*T* = 295 K
0.40 × 0.35 × 0.20 mm

#### Data collection

Oxford Diffraction Gemini R Ultra Ruby CCD diffractometer
21109 measured reflections
3914 independent reflections
2956 reflections with *I* > 2σ(*I*)
*R*_int_ = 0.039

#### Refinement

*R*[*F* ^2^ > 2σ(*F* ^2^)] = 0.039
*wR*(*F* ^2^) = 0.116
*S* = 1.10
3914 reflections
309 parameters
H-atom parameters constrained
Δρ_max_ = 0.20 e Å^−3^
Δρ_min_ = −0.30 e Å^−3^

### 

Data collection: *CrysAlis CCD* (Oxford Diffraction, 2008 ▶); cell refinement: *CrysAlis RED* (Oxford Diffraction, 2008 ▶); data reduction: *CrysAlis RED*; program(s) used to solve structure: *SHELXS97* (Sheldrick, 2008 ▶); program(s) used to refine structure: *SHELXL97* (Sheldrick, 2008 ▶); molecular graphics: *ORTEP-3* (Farrugia, 1997 ▶); software used to prepare material for publication: *SHELXL97* (Sheldrick, 2008 ▶) and *PLATON* (Spek, 2009 ▶).

## Supplementary Material

Crystal structure: contains datablocks global, I. DOI: 10.1107/S1600536810008950/ng2739sup1.cif

Structure factors: contains datablocks I. DOI: 10.1107/S1600536810008950/ng2739Isup2.hkl

Additional supplementary materials:  crystallographic information; 3D view; checkCIF report

## Figures and Tables

**Table 1:** Hydrogen-bond geometry (Å, °) *Cg*4 is the centroid of the C18–C23 ring.

*D*—H⋯*A*	*D*—H	H⋯*A*	*D*⋯*A*	*D*—H⋯*A*
C2—H2⋯O28^i^	0.93	2.55	3.221 (2)	130
C5—H5⋯O28^ii^	0.93	2.56	3.222 (3)	129
C24—H24*B*⋯*Cg*4^iii^	0.96	2.92	3.603 (2)	129
C26—H26*A*⋯O29^ii^	0.96	2.43	3.280 (3)	148
C26—H26*C*⋯*Cg*4^ii^	0.96	2.80	3.741 (2)	165

Symmetry codes: (i) 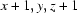; (ii) 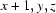; (iii) 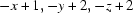.

**Table 2:** C—F⋯π inter­actions (Å,°) *Cg*1 and *Cg*3 are the centroids of the C9/N10/C11–C14 and C5–C8/C13/C14 rings, respectively.

*X*—*I*⋯*J*	*I*⋯*J*	*X*⋯*J*	*X*—*I*⋯*J*
C31—F32⋯*Cg*3^iv^	3.474 (2)	4.003 (2)	103.67 (14)
C31—F33⋯*Cg*1^iv^	3.241 (2)	4.087 (2)	121.73 (14)
C31—F34⋯*Cg*3^iv^	3.762 (2)	4.003 (2)	90.62 (13)

Symmetry code: (iv) −*x* + 1, −*y* + 2, −*z* + 1.

**Table 3:** π–π inter­actions (Å,°) *Cg*1, *Cg*2 and *Cg*3 are the centroids of the C9/N10/C11–C14, C1–C4/C11/C12 and C5–C8/C13/C14 rings, respectively. *CgI*⋯*CgJ* is the distance between ring centroids. The dihedral angle is that between the planes of the rings *I* and *J. CgI*_Perp is the perpendicular distance of *CgI* from ring *J. CgI*_Offset is the distance between *CgI* and perpendicular projection of *CgJ* on ring *I*.

*I*	*J*	*CgI*⋯*CgJ*	Dihedral angle	*CgI*_Perp	*CgI*_Offset
1	1^v^	4.022 (2)	0.00	3.571 (2)	1.850 (2)
1	3^v^	3.702 (2)	1.80	3.532 (2)	1.109 (2)
2	3^v^	3.965 (2)	4.29	3.451 (2)	1.960 (2)
3	1^v^	3.702 (2)	1.80	3.544 (2)	1.070 (2)
3	2^v^	3.965 (2)	4.29	3.566 (2)	1.733 (2)

Symmetry code: (v) −*x* + 1, −*y* + 1, −*z* + 1.

**Fig. 1.**
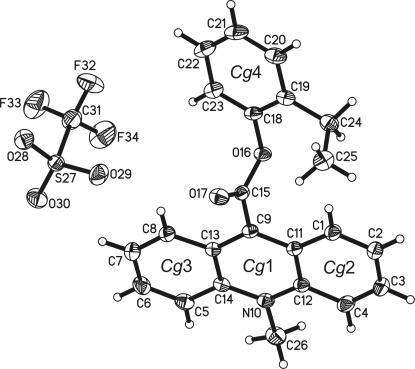
The molecular structure of the title compound showing the atom-labeling scheme. Displacement ellipsoids are drawn at the 25% probability level and H atoms are shown as small spheres of arbitrary radius. Cg1, Cg2, Cg3 and Cg4 denote the ring centroids.

**Fig. 2.**
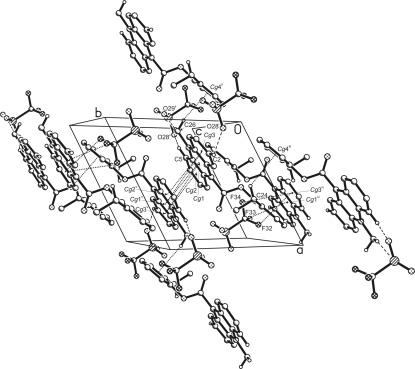
The arrangement of the ions in the crystal structure. The C–H···O interactions are represented by dashed lines, the C–H···π, C–F···π and π–π contacts by dotted lines. H atoms not involved in interactions have been omitted. [Symmetry codes: (i) x + 1, y, z + 1; (ii) x + 1, y, z; (iii) –x + 1, –y + 2, –z + 2; (iv) –x + 1, –y + 2, –z + 1; (v) –x + 1, –y + 1, –z + 1.]

## supplementary crystallographic information

### Comment

9-(Phenoxycarbonyl)-10-alkylacridinium salts have long been known as chemiluminescent indicators or the chemiluminogenic fragments of chemiluminescent labels (Zomer & Jacquemijns, 2001). These compounds are commonly applied in assays of biologically and environmentally important entities such as antigens, antibodies, enzymes or DNA fragments (Roda *et al.*, 2003; Brown *et al.*, 2009). The reaction of the cations of these salts with hydrogen peroxide in alkaline media produces light. Our own investigations (Rak *et al.*, 1999) and those of others (Zomer *et al.*, 2001) have revealed that oxidation of acridinium chemiluminogens is accompanied by the removal of the phenoxycarbonyl fragment and the conversion of the remaining molecules to electronically excited, light-emitting 10-alkyl-9-acridinones. It has been found that the efficiency of chemiluminescence – crucial for analytical applications – is affected by the constitution of the phenyl fragment (Zomer & Jacquemijns, 2001). In the search for efficient chemiluminogens we undertook investigations on 9-(phenoxycarbonyl)-10-methylacridinium derivatives substituted in the phenyl fragment. Here we present the structure of one such derivative.

In the cation of the title compound (Fig. 1), the bond lengths and angles characterizing the geometry of the acridinium moiety are typical of acridine-based derivatives (Sikorski *et al.*, 2005*a*,*b*). With respective average deviations from planarity of 0.022 (3) Å and 0.002 (3) Å, the acridine and benzene ring systems are oriented at 20.8 (1)°. The carboxyl group is twisted at an angle of 66.2 (1)° relative to the acridine skeleton. The mean planes of the adjacent acridine moieties are parallel (remain at an angle of 0.0 (1)°) in the lattice. The mutual arrangement of the carboxyl group relative to the acridine skeleton is similar in the compound investigated and its precursor – 2-ethylphenyl acridine-9-carboxylate (Sikorski *et al.*, 2005*a*). On the other hand, the acridine and benzene ring systems are oriented quite differently in the compound investigated and its precursor.

In the crystal structure, the inversely oriented cations form dimers through multidirectional π–π interactions involving acridine moieties (Table 3, Fig. 2). These dimers are linked by C–H···O (Table 1, Fig. 2) and C–F···π (Table 2, Fig. 2) interactions to adjacent anions, and by C–H···π (Table 1, Fig. 2) interactions to neighboring cations. The C–H···O interactions are of the hydrogen bond type (Steiner, 1999; Bianchi *et al.* 2004). The C–H···π interactions should be of an attractive nature (Takahashi *et al.*, 2001), like the C–F···π (Dorn *et al.*, 2005) and the π–π (Hunter *et al.*, 2001) interactions. The crystal structure is stabilized by a network of these short-range specific interactions and by long-range electrostatic interactions between ions.

### Experimental

The compound was synthesized in three steps (Niziołek *et al.*, 2008). First, 9-(chlorocarbonyl)-acridine was produced by treating acridine-9-carboxylic acid with a tenfold molar excess of thionyl chloride. Then, esterification  with 2-ethylphenol was carried out in anhydrous dichloromethane in the presence of *N*,*N*-diethylethanamine and a catalytic amount of *N*,*N*-dimethyl-4-pyridinamine (room temperature, 15h) (Sato, 1996). The crude product was purified chromatographically (SiO_2_, cyclohexane/ethyl acetate, 3/2 v/v). The 2-ethylphenyl acridine-9-carboxylate thus obtained was quaternarized with a five-fold molar excess of methyl trifluoromethanesulfonate dissolved in anhydrous dichloromethane. The crude 9-(2-ethylphenoxycarbonyl)-10-methylacridinium trifluoromethanesulfonate was dissolved in a small amount of ethanol, filtered and precipitated with a 25 v/v excess of diethyl ether. Yellow crystals suitable suitable for X-Ray investigations were grown from absolute ethanol solution (m.p. 470-471 K).

### Refinement

H atoms were positioned geometrically, with C—H = 0.93 Å and 0.96 Å for the aromatic and alkyl H atoms, respectively, and constrained to ride on their parrent atoms with *U*_iso_(H) = *xU*_eq_(C), where *x* = 1.2 for the aromatic and *x* = 1.5 for the alkyl H atoms.

### Crystal data

**Table:**

C_23_H_20_NO_2_^+^·CF_3_O_3_S^−^	*Z* = 2
*M**_r_* = 491.47	*F*(000) = 508
Triclinic, *P*1	*D*_x_ = 1.484 Mg m^−^^3^
Hall symbol: -P 1	Mo *K*α radiation, λ = 0.71073 Å
*a* = 9.8519 (4) Å	Cell parameters from 10425 reflections
*b* = 10.9533 (4) Å	θ = 3.1–29.2°
*c* = 11.7805 (4) Å	µ = 0.21 mm^−^^1^
α = 104.379 (3)°	*T* = 295 K
β = 101.475 (3)°	Block, yellow
γ = 109.983 (3)°	0.40 × 0.35 × 0.20 mm
*V* = 1099.61 (7) Å^3^	

### Data collection

**Table:**

Oxford Diffraction Gemini R Ultra Ruby CCD diffractometer	2956 reflections with *I* > 2σ(*I*)
Radiation source: Enhanced (Mo) X-ray Source	*R*_int_ = 0.039
graphite	θ_max_ = 25.1°, θ_min_ = 3.1°
Detector resolution: 10.4002 pixels mm^-1^	*h* = −11→11
ω scans	*k* = −13→13
21109 measured reflections	*l* = −14→14
3914 independent reflections	

### Refinement

**Table:**

Refinement on *F*^2^	Primary atom site location: structure-invariant direct methods
Least-squares matrix: full	Secondary atom site location: difference Fourier map
*R*[*F*^2^ > 2σ(*F*^2^)] = 0.039	Hydrogen site location: inferred from neighbouring sites
*wR*(*F*^2^) = 0.116	H-atom parameters constrained
*S* = 1.10	*w* = 1/[σ^2^(*F*_o_^2^) + (0.0737*P*)^2^] where *P* = (*F*_o_^2^ + 2*F*_c_^2^)/3
3914 reflections	(Δ/σ)_max_ < 0.001
309 parameters	Δρ_max_ = 0.20 e Å^−^^3^
0 restraints	Δρ_min_ = −0.30 e Å^−^^3^

### Special details

**Table:**

Geometry. All esds (except the esd in the dihedral angle between two l.s. planes) are estimated using the full covariance matrix. The cell esds are taken into account individually in the estimation of esds in distances, angles and torsion angles; correlations between esds in cell parameters are only used when they are defined by crystal symmetry. An approximate (isotropic) treatment of cell esds is used for estimating esds involving l.s. planes.

### Fractional atomic coordinates and isotropic or equivalent isotropic displacement parameters (Å^2^)

**Table:**

	*x*	*y*	*z*	*U*_iso_*/*U*_eq_	
C8	0.4629 (2)	0.70220 (17)	0.44477 (16)	0.0468 (4)	
H8	0.3781	0.7136	0.4600	0.056*	
C7	0.4857 (2)	0.69994 (19)	0.33477 (18)	0.0561 (5)	
H7	0.4164	0.7087	0.2745	0.067*	
C6	0.6146 (3)	0.68432 (19)	0.31214 (18)	0.0573 (5)	
H6	0.6298	0.6842	0.2367	0.069*	
C5	0.7175 (2)	0.66940 (17)	0.39617 (17)	0.0508 (5)	
H5	0.8014	0.6591	0.3780	0.061*	
C4	0.8753 (2)	0.62222 (19)	0.79550 (18)	0.0535 (5)	
H4	0.9549	0.6038	0.7759	0.064*	
C3	0.8550 (2)	0.6234 (2)	0.90569 (19)	0.0583 (5)	
H3	0.9214	0.6064	0.9612	0.070*	
C2	0.7358 (2)	0.64984 (19)	0.93822 (18)	0.0559 (5)	
H2	0.7247	0.6518	1.0151	0.067*	
C1	0.6372 (2)	0.67241 (18)	0.85755 (16)	0.0499 (5)	
H1	0.5573	0.6881	0.8792	0.060*	
C9	0.55076 (18)	0.69321 (15)	0.65368 (15)	0.0374 (4)	
N10	0.79811 (15)	0.65304 (13)	0.59893 (13)	0.0419 (3)	
C13	0.56732 (19)	0.68738 (15)	0.53759 (15)	0.0381 (4)	
C14	0.69716 (19)	0.66950 (15)	0.51165 (15)	0.0403 (4)	
C11	0.65277 (19)	0.67276 (16)	0.74035 (15)	0.0394 (4)	
C12	0.77656 (19)	0.64875 (15)	0.70957 (15)	0.0404 (4)	
C15	0.4184 (2)	0.71774 (16)	0.68401 (15)	0.0389 (4)	
O16	0.46656 (13)	0.83893 (11)	0.77566 (10)	0.0435 (3)	
O17	0.28868 (14)	0.63985 (12)	0.63199 (12)	0.0554 (4)	
C18	0.3528 (2)	0.88341 (16)	0.80457 (16)	0.0439 (4)	
C19	0.3118 (2)	0.86926 (16)	0.90748 (17)	0.0478 (4)	
C20	0.2093 (2)	0.92574 (19)	0.9350 (2)	0.0615 (6)	
H20	0.1775	0.9189	1.0032	0.074*	
C21	0.1550 (3)	0.9905 (2)	0.8641 (2)	0.0690 (6)	
H21	0.0875	1.0273	0.8851	0.083*	
C22	0.1986 (3)	1.0024 (2)	0.7621 (2)	0.0671 (6)	
H22	0.1602	1.0460	0.7140	0.080*	
C23	0.3001 (2)	0.94881 (18)	0.73174 (19)	0.0552 (5)	
H23	0.3321	0.9567	0.6637	0.066*	
C24	0.3714 (2)	0.79938 (18)	0.98693 (17)	0.0554 (5)	
H24A	0.3664	0.8357	1.0692	0.067*	
H24B	0.4774	0.8221	0.9933	0.067*	
C25	0.2850 (3)	0.6427 (2)	0.9388 (2)	0.0644 (5)	
H25A	0.3346	0.6040	0.9891	0.097*	
H25B	0.2832	0.6063	0.8552	0.097*	
H25C	0.1828	0.6189	0.9416	0.097*	
C26	0.9362 (2)	0.6389 (2)	0.5764 (2)	0.0623 (5)	
H26A	0.9481	0.6594	0.5034	0.093*	
H26B	0.9257	0.5459	0.5655	0.093*	
H26C	1.0239	0.7022	0.6456	0.093*	
S27	0.07676 (5)	0.73990 (4)	0.26699 (4)	0.04742 (17)	
O28	−0.07260 (15)	0.73377 (15)	0.22221 (13)	0.0649 (4)	
O29	0.09669 (19)	0.68219 (15)	0.36225 (14)	0.0740 (4)	
O30	0.14440 (18)	0.70502 (16)	0.17469 (13)	0.0743 (4)	
C31	0.1906 (3)	0.9229 (2)	0.3455 (2)	0.0740 (6)	
F32	0.1421 (2)	0.97409 (17)	0.43606 (15)	0.1282 (7)	
F33	0.1861 (2)	0.99425 (14)	0.27126 (17)	0.1192 (6)	
F34	0.33510 (19)	0.95023 (16)	0.39672 (18)	0.1263 (7)	

### Atomic displacement parameters (Å^2^)

**Table:**

	*U*^11^	*U*^22^	*U*^33^	*U*^12^	*U*^13^	*U*^23^
C8	0.0487 (11)	0.0493 (10)	0.0471 (10)	0.0242 (9)	0.0164 (9)	0.0174 (8)
C7	0.0650 (14)	0.0608 (11)	0.0476 (11)	0.0298 (10)	0.0164 (10)	0.0226 (9)
C6	0.0724 (14)	0.0590 (11)	0.0465 (11)	0.0259 (10)	0.0281 (10)	0.0219 (9)
C5	0.0521 (12)	0.0501 (10)	0.0548 (11)	0.0202 (9)	0.0292 (10)	0.0168 (8)
C4	0.0414 (11)	0.0578 (11)	0.0627 (12)	0.0277 (9)	0.0110 (9)	0.0169 (9)
C3	0.0565 (13)	0.0641 (12)	0.0565 (12)	0.0317 (10)	0.0074 (10)	0.0226 (9)
C2	0.0645 (13)	0.0650 (12)	0.0485 (11)	0.0358 (11)	0.0167 (10)	0.0240 (9)
C1	0.0541 (12)	0.0592 (11)	0.0501 (10)	0.0328 (9)	0.0219 (9)	0.0233 (9)
C9	0.0346 (9)	0.0342 (8)	0.0447 (9)	0.0151 (7)	0.0147 (7)	0.0123 (7)
N10	0.0325 (8)	0.0425 (7)	0.0491 (8)	0.0162 (6)	0.0158 (7)	0.0096 (6)
C13	0.0388 (9)	0.0344 (8)	0.0409 (9)	0.0157 (7)	0.0134 (7)	0.0109 (7)
C14	0.0402 (10)	0.0351 (8)	0.0429 (9)	0.0134 (7)	0.0168 (8)	0.0090 (7)
C11	0.0379 (10)	0.0372 (8)	0.0438 (9)	0.0171 (7)	0.0133 (8)	0.0125 (7)
C12	0.0354 (9)	0.0366 (8)	0.0447 (10)	0.0145 (7)	0.0107 (8)	0.0086 (7)
C15	0.0401 (11)	0.0419 (9)	0.0410 (9)	0.0212 (8)	0.0151 (8)	0.0166 (7)
O16	0.0375 (7)	0.0442 (6)	0.0504 (7)	0.0205 (5)	0.0174 (6)	0.0106 (5)
O17	0.0375 (8)	0.0531 (7)	0.0640 (8)	0.0168 (6)	0.0143 (6)	0.0053 (6)
C18	0.0367 (10)	0.0373 (8)	0.0559 (11)	0.0181 (7)	0.0156 (8)	0.0079 (8)
C19	0.0435 (10)	0.0404 (9)	0.0545 (11)	0.0151 (8)	0.0194 (9)	0.0082 (8)
C20	0.0549 (13)	0.0570 (11)	0.0730 (13)	0.0250 (10)	0.0312 (11)	0.0116 (10)
C21	0.0579 (14)	0.0588 (12)	0.0956 (17)	0.0356 (11)	0.0316 (13)	0.0131 (12)
C22	0.0613 (14)	0.0552 (11)	0.0932 (16)	0.0354 (11)	0.0211 (12)	0.0257 (11)
C23	0.0530 (12)	0.0502 (10)	0.0668 (12)	0.0251 (9)	0.0205 (10)	0.0203 (9)
C24	0.0553 (12)	0.0599 (11)	0.0536 (11)	0.0243 (10)	0.0252 (10)	0.0163 (9)
C25	0.0621 (14)	0.0642 (12)	0.0767 (14)	0.0260 (10)	0.0307 (11)	0.0335 (11)
C26	0.0382 (11)	0.0780 (13)	0.0666 (13)	0.0265 (10)	0.0203 (10)	0.0117 (11)
S27	0.0507 (3)	0.0533 (3)	0.0460 (3)	0.0255 (2)	0.0210 (2)	0.0191 (2)
O28	0.0476 (9)	0.0797 (9)	0.0687 (9)	0.0282 (7)	0.0186 (7)	0.0245 (7)
O29	0.0943 (12)	0.0861 (10)	0.0733 (10)	0.0504 (9)	0.0386 (9)	0.0506 (8)
O30	0.0806 (11)	0.1006 (11)	0.0617 (9)	0.0510 (9)	0.0407 (8)	0.0266 (8)
C31	0.0712 (17)	0.0610 (13)	0.0783 (15)	0.0277 (12)	0.0042 (13)	0.0196 (12)
F32	0.1628 (18)	0.1019 (12)	0.0962 (11)	0.0735 (12)	0.0201 (12)	−0.0158 (9)
F33	0.1130 (13)	0.0720 (9)	0.1535 (15)	0.0184 (9)	0.0082 (11)	0.0619 (10)
F34	0.0701 (11)	0.0882 (10)	0.1607 (16)	0.0141 (9)	−0.0251 (11)	0.0193 (10)

### Geometric parameters (Å, °)

**Table:**

C8—C7	1.354 (3)	O16—C18	1.432 (2)
C8—C13	1.427 (2)	C18—C23	1.379 (2)
C8—H8	0.9300	C18—C19	1.380 (3)
C7—C6	1.405 (3)	C19—C20	1.401 (3)
C7—H7	0.9300	C19—C24	1.500 (3)
C6—C5	1.352 (3)	C20—C21	1.365 (3)
C6—H6	0.9300	C20—H20	0.9300
C5—C14	1.414 (2)	C21—C22	1.375 (3)
C5—H5	0.9300	C21—H21	0.9300
C4—C3	1.350 (3)	C22—C23	1.382 (3)
C4—C12	1.416 (3)	C22—H22	0.9300
C4—H4	0.9300	C23—H23	0.9300
C3—C2	1.402 (3)	C24—C25	1.523 (3)
C3—H3	0.9300	C24—H24A	0.9700
C2—C1	1.349 (3)	C24—H24B	0.9700
C2—H2	0.9300	C25—H25A	0.9600
C1—C11	1.420 (2)	C25—H25B	0.9600
C1—H1	0.9300	C25—H25C	0.9600
C9—C13	1.398 (2)	C26—H26A	0.9600
C9—C11	1.401 (2)	C26—H26B	0.9600
C9—C15	1.509 (2)	C26—H26C	0.9600
N10—C12	1.371 (2)	S27—O30	1.4242 (14)
N10—C14	1.374 (2)	S27—O29	1.4307 (14)
N10—C26	1.488 (2)	S27—O28	1.4331 (15)
C13—C14	1.437 (2)	S27—C31	1.806 (2)
C11—C12	1.427 (2)	C31—F33	1.314 (3)
C15—O17	1.192 (2)	C31—F34	1.326 (3)
C15—O16	1.3442 (19)	C31—F32	1.330 (3)
			
C7—C8—C13	120.82 (17)	C23—C18—O16	116.69 (16)
C7—C8—H8	119.6	C19—C18—O16	119.15 (16)
C13—C8—H8	119.6	C18—C19—C20	115.51 (18)
C8—C7—C6	119.67 (19)	C18—C19—C24	123.48 (16)
C8—C7—H7	120.2	C20—C19—C24	121.01 (18)
C6—C7—H7	120.2	C21—C20—C19	121.7 (2)
C5—C6—C7	122.33 (18)	C21—C20—H20	119.1
C5—C6—H6	118.8	C19—C20—H20	119.1
C7—C6—H6	118.8	C20—C21—C22	120.97 (19)
C6—C5—C14	119.89 (18)	C20—C21—H21	119.5
C6—C5—H5	120.1	C22—C21—H21	119.5
C14—C5—H5	120.1	C21—C22—C23	119.4 (2)
C3—C4—C12	120.51 (18)	C21—C22—H22	120.3
C3—C4—H4	119.7	C23—C22—H22	120.3
C12—C4—H4	119.7	C18—C23—C22	118.5 (2)
C4—C3—C2	121.39 (18)	C18—C23—H23	120.8
C4—C3—H3	119.3	C22—C23—H23	120.8
C2—C3—H3	119.3	C19—C24—C25	113.77 (17)
C1—C2—C3	119.77 (19)	C19—C24—H24A	108.8
C1—C2—H2	120.1	C25—C24—H24A	108.8
C3—C2—H2	120.1	C19—C24—H24B	108.8
C2—C1—C11	121.50 (18)	C25—C24—H24B	108.8
C2—C1—H1	119.2	H24A—C24—H24B	107.7
C11—C1—H1	119.2	C24—C25—H25A	109.5
C13—C9—C11	120.83 (15)	C24—C25—H25B	109.5
C13—C9—C15	119.28 (15)	H25A—C25—H25B	109.5
C11—C9—C15	119.87 (15)	C24—C25—H25C	109.5
C12—N10—C14	121.94 (14)	H25A—C25—H25C	109.5
C12—N10—C26	117.26 (16)	H25B—C25—H25C	109.5
C14—N10—C26	120.80 (15)	N10—C26—H26A	109.5
C9—C13—C8	122.77 (15)	N10—C26—H26B	109.5
C9—C13—C14	118.70 (16)	H26A—C26—H26B	109.5
C8—C13—C14	118.51 (15)	N10—C26—H26C	109.5
N10—C14—C5	121.66 (16)	H26A—C26—H26C	109.5
N10—C14—C13	119.56 (15)	H26B—C26—H26C	109.5
C5—C14—C13	118.77 (17)	O30—S27—O29	114.62 (9)
C9—C11—C1	122.87 (16)	O30—S27—O28	115.38 (9)
C9—C11—C12	119.01 (15)	O29—S27—O28	115.01 (9)
C1—C11—C12	118.12 (16)	O30—S27—C31	103.15 (11)
N10—C12—C4	121.62 (16)	O29—S27—C31	103.45 (10)
N10—C12—C11	119.71 (15)	O28—S27—C31	102.78 (10)
C4—C12—C11	118.67 (16)	F33—C31—F34	107.8 (2)
O17—C15—O16	125.00 (15)	F33—C31—F32	106.5 (2)
O17—C15—C9	123.94 (15)	F34—C31—F32	106.5 (2)
O16—C15—C9	111.05 (14)	F33—C31—S27	112.23 (16)
C15—O16—C18	117.14 (13)	F34—C31—S27	112.05 (16)
C23—C18—C19	123.94 (16)	F32—C31—S27	111.42 (18)
			
C13—C8—C7—C6	−0.7 (3)	C9—C11—C12—N10	3.3 (2)
C8—C7—C6—C5	0.9 (3)	C1—C11—C12—N10	−177.78 (14)
C7—C6—C5—C14	−0.1 (3)	C9—C11—C12—C4	−177.29 (15)
C12—C4—C3—C2	0.4 (3)	C1—C11—C12—C4	1.6 (2)
C4—C3—C2—C1	1.1 (3)	C13—C9—C15—O17	63.9 (2)
C3—C2—C1—C11	−1.2 (3)	C11—C9—C15—O17	−114.36 (19)
C11—C9—C13—C8	177.50 (15)	C13—C9—C15—O16	−115.39 (16)
C15—C9—C13—C8	−0.7 (2)	C11—C9—C15—O16	66.40 (18)
C11—C9—C13—C14	−4.3 (2)	O17—C15—O16—C18	−6.3 (2)
C15—C9—C13—C14	177.54 (13)	C9—C15—O16—C18	172.96 (13)
C7—C8—C13—C9	178.04 (16)	C15—O16—C18—C23	−82.25 (18)
C7—C8—C13—C14	−0.2 (2)	C15—O16—C18—C19	102.93 (18)
C12—N10—C14—C5	−177.94 (14)	C23—C18—C19—C20	0.5 (3)
C26—N10—C14—C5	2.0 (2)	O16—C18—C19—C20	174.87 (14)
C12—N10—C14—C13	2.1 (2)	C23—C18—C19—C24	−179.24 (17)
C26—N10—C14—C13	−178.01 (14)	O16—C18—C19—C24	−4.8 (2)
C6—C5—C14—N10	179.25 (16)	C18—C19—C20—C21	−0.2 (3)
C6—C5—C14—C13	−0.8 (2)	C24—C19—C20—C21	179.50 (18)
C9—C13—C14—N10	2.6 (2)	C19—C20—C21—C22	0.3 (3)
C8—C13—C14—N10	−179.08 (14)	C20—C21—C22—C23	−0.6 (3)
C9—C13—C14—C5	−177.38 (14)	C19—C18—C23—C22	−0.8 (3)
C8—C13—C14—C5	0.9 (2)	O16—C18—C23—C22	−175.31 (16)
C13—C9—C11—C1	−177.50 (15)	C21—C22—C23—C18	0.8 (3)
C15—C9—C11—C1	0.7 (2)	C18—C19—C24—C25	−83.8 (2)
C13—C9—C11—C12	1.4 (2)	C20—C19—C24—C25	96.5 (2)
C15—C9—C11—C12	179.56 (13)	O30—S27—C31—F33	60.3 (2)
C2—C1—C11—C9	178.74 (17)	O29—S27—C31—F33	−179.97 (18)
C2—C1—C11—C12	−0.1 (3)	O28—S27—C31—F33	−60.0 (2)
C14—N10—C12—C4	175.56 (15)	O30—S27—C31—F34	−61.1 (2)
C26—N10—C12—C4	−4.4 (2)	O29—S27—C31—F34	58.6 (2)
C14—N10—C12—C11	−5.0 (2)	O28—S27—C31—F34	178.62 (18)
C26—N10—C12—C11	175.04 (14)	O30—S27—C31—F32	179.70 (16)
C3—C4—C12—N10	177.62 (16)	O29—S27—C31—F32	−60.58 (18)
C3—C4—C12—C11	−1.8 (3)	O28—S27—C31—F32	59.41 (18)

### Hydrogen-bond geometry (Å, °)

**Table:**

*Cg*4 is the centroid of the C18–C23 ring.

**Table:**

*D*—H···*A*	*D*—H	H···*A*	*D*···*A*	*D*—H···*A*
C2—H2···O28^i^	0.93	2.55	3.221 (2)	130
C5—H5···O28^ii^	0.93	2.56	3.222 (3)	129
C24—H24B···*Cg*4^iii^	0.96	2.92	3.603 (2)	129
C26—H26A···O29^ii^	0.96	2.43	3.280 (3)	148
C26—H26C···*Cg*4^ii^	0.96	2.80	3.741 (2)	165

Symmetry codes:  (i) *x*+1, *y*, *z*+1; (ii) *x*+1, *y*, *z*; (iii) −*x*+1, −*y*+2, −*z*+2.

### Table 2 C—F···π interactions (Å,°)

*Cg*1 and *Cg*3 are the centroids of the C9/N10/C11–C14 and C5–C8/C13/C14 rings, respectively.

**Table:**

*X*—*I*···*J*	*I*···*J*	*X*···*J*	*X*—*I*···*J*
C31—F32···*Cg*3^iv^	3.474 (2)	4.003 (2)	103.67 (14)
C31—F33···*Cg*1^iv^	3.241 (2)	4.087 (2)	121.73 (14)
C31—F34···*Cg*3^iv^	3.762 (2)	4.003 (2)	90.62 (13)

Symmetry code: (iv) -x + 1, -y+ 2, -z + 1.

### Table 3 π–π interactions (Å,°)

*Cg*1, *Cg*2 and *Cg*3 are the centroids of the C9/N10/C11–C14, C1–C4/C11/C12 and C5–C8/C13/C14 rings, respectively. *CgI*···*CgJ* is the distance between ring centroids. The dihedral angle is that between the planes of the rings *I* and *J*. *CgI*_Perp is the perpendicular distance of *CgI* from ring *J*. *CgI*_Offset is the distance between *CgI* and perpendicular projection of *CgJ* on ring *I*.

**Table:**

*I*	*J*	*CgI*···*CgJ*	Dihedral angle	*CgI*_Perp	*CgI*_Offset
1	1^v^	4.022 (2)	0.00	3.571 (2)	1.850 (2)
1	3^v^	3.702 (2)	1.80	3.532 (2)	1.109 (2)
2	3^v^	3.965 (2)	4.29	3.451 (2)	1.960 (2)
3	1^v^	3.702 (2)	1.80	3.544 (2)	1.070 (2)
3	2^v^	3.965 (2)	4.29	3.566 (2)	1.733 (2)

Symmetry code: (v) -x + 1, -y + 1, -z + 1.
